# Linear and Nonlinear Deformation Effects in the Permanent GNSS Network of Cyprus

**DOI:** 10.3390/s20061768

**Published:** 2020-03-22

**Authors:** Chris Danezis, Miltiadis Chatzinikos, Christopher Kotsakis

**Affiliations:** 1 Department of Civil Engineering and Geomatics, Cyprus University of Technology, Limassol 3036, Cyprus; 2 ERATOSTHENES Centre of Excellence, Limassol 3036, Cyprus; 3 Department of Geodesy and Surveying, Aristotle University of Thessaloniki, 54124 Thessaloniki, Greece; mchatzin@topo.auth.gr (M.C.); kotsaki@topo.auth.gr (C.K.)

**Keywords:** GNSS, CORS, crustal deformation, seasonal variations, station velocities, position timeseries, Cyprus

## Abstract

Permanent Global Navigation Satellite Systems (GNSS) reference stations are well established as a powerful tool for the estimation of deformation induced by man-made or physical processes. GNSS sensors are successfully used to determine positions and velocities over a specified time period, with unprecedented accuracy, promoting research in many safety-critical areas, such as geophysics and geo-tectonics, tackling problems that torment traditional equipment and providing deformation products with absolute accuracy. Cyprus, being located at the Mediterranean fault, exhibits a very interesting geodynamic regime, which has yet to be investigated thoroughly. Accordingly, this research revolves around the estimation of crustal deformation in Cyprus using GNSS receivers. CYPOS (CYprus POsitioning System), a network of seven permanent GNSS stations has been operating since 2008, under the responsibility of the Department of Lands and Surveys. The continuous flow of positioning data collected over this network, offers the required information to investigate the behavior of the crustal deformation field of Cyprus using GNSS sensors for the first time. This paper presents the results of a multi-year analysis (11/2011–01/2017) of daily GNSS data and provides inferences of linear and nonlinear deforming signals into the position time series of the network stations. Specifically, 3D station velocities and seasonal periodic displacements are jointly estimated and presented via a data stacking approach with respect to the IGb08 reference frame.

## 1. Introduction

The use of permanent Global Navigation Satellite Systems (GNSS) networks offers the potential to continuously observe the motion of Earth-fixed stations. This enables precise monitoring of crustal displacements through the analysis of position time series derived from daily or weekly GNSS network adjustments under identical processing options for each session solution. In general, the temporal variations that appear in the position time series of permanent GNSS stations, either in the horizontal or vertical spatial components, are associated with four main sources:

- True Earth deformation due to various geophysical phenomena, such as lithospheric tectonic displacements in global or regional scale [1,2,3,4,5], post-glacial rebound [6,7,8,9], atmospheric pressure, hydrological and ocean loading effects [10,11,12,13,14,15];
- GNSS-related observational or modeling errors, like multipath [11,16], orbital errors [17] and inefficient atmospheric models [18];
- Sudden geophysical or non-geophysical events, such as position discontinuities due to antenna change [19,20], antenna calibration model switch [21] and co-seismic displacements [22,23];
- Temporal systematic disturbances of the reference frame that is used in the description of position time series [24,25].

Two of the most important and strongest signals that are regularly modeled in the analysis of GNSS position time series is (a) their long-term (secular) linear trend due to constant station velocities, and (b) their nonlinear evolution due to the presence of seasonal (periodic or quasi-periodic) variations with constant or time-dependent amplitude and phase. The linear signal is of great interest for geodynamical studies as it represents the dominating effect caused by tectonic plate motion and post-glacial rebound on the horizontal and vertical station displacements, respectively [5]. The Euler pole model with constant angular velocity is a common tool for describing the horizontal part of secular displacements as a circular motion of plate-fixed points over the Earth’s surface [26,27]. Within the limited time span of few decades, the circular plate motion is sufficiently approximated in terms of constant station velocities with respect to a Cartesian terrestrial reference frame such as the International Terrestrial Reference Frame (ITRF) [2,28]. On the other hand, the nonlinear part in GNSS position time series originating from seasonal periodic signals is not totally understood [12,13], yet a significant portion should be attributed to the influence of Earth loading effects that remain unmodeled during the GNSS data processing [14,15]. Note that such seasonal variations do not always have a strict periodic character due to disturbances in their associated geophysical sources within the dynamic Earth system. 

The scope of this paper is to present the results from the daily and multi-year processing of continuous GNSS data at the seven permanent GNSS stations of CYPOS (CYprus POsitioning System). These stations are described in detail in Section 2.1 and they currently provide the skeleton of the national geodetic network for GNSS-based applications in Cyprus. The analysis of linear and non-linear temporal variations in their position time series, which is performed herein for the first time, will provide useful insight on the deformation characteristics of CYPOS.

The main outcome of this work consists of: (a) the estimated geocentric Cartesian coordinates (at epoch $t_{0}=2005.0$) and velocities of the CYPOS GNSS stations with respect to the IGb08 frame [29], and (b) their residual position time series, after removing the velocity-based secular trends, with respect to the local topocentric system of each station (East, North, Up components). The choice of the global GNSS-based frame IGb08, instead of ITRF2008, is made to ensure maximum consistency with the precise geodetic products (satellite orbits, clocks) that were used during the GNSS data processing. Both of the above outcomes have been further analyzed to infer the local deformation field in the network of CYPOS and the presence of seasonal signals in the respective time series of the network stations. The main findings are presented in the following sections of this research in detail.

## 2. Processing of Continuous GNSS Data

### 2.1. GNSS Permanent Stations in Cyprus

In general, GNSS Continuously Operating Reference Stations (CORS) stations can be divided in three main categories [30]:

Tier-1 CORS are stations used by ultra-high accuracy networks, such as the International GNSS Service (IGS) or the EUREF Permanent Network (EPN) to enable geophysical research or support the definition of global or continental reference frames. Consequently, the IGS and EPN stations are primarily used to define the ITRF, and the European Terrestrial Reference Frame (ETRF) respectively. Their monumentation is carried out under stringent specifications to achieve the highest degree of stability [31].

Tier-2 CORS are stations that require an equivalent degree of monument stability and are usually established by national geodetic agencies to realize and maintain national geodetic reference frames. Note that these CORS form the primary national Global Positioning System (GPS)/GNSS network, and that Tier-1 CORS may usually be a subset of Tier-2 stations to provide a tie between the national geodetic datum and the ITRF or ETRF.

Finally, Tier-3 CORS require stable monuments (less stable designs than the previous two tiers may be accepted) and are established by national, state, territory governments, and/or commercial agencies to densify the national infrastructure and to offer real-time positioning applications (Differential GNSS or Real Time Kinematic services). Usually, Tier-3 stations operate as part of the national datum, rather than define it. In such cases, the interstation distance required to achieve centimeter-level positioning accuracy (by means of real-time services) is around 70 Km [32].

**Figure 1:**
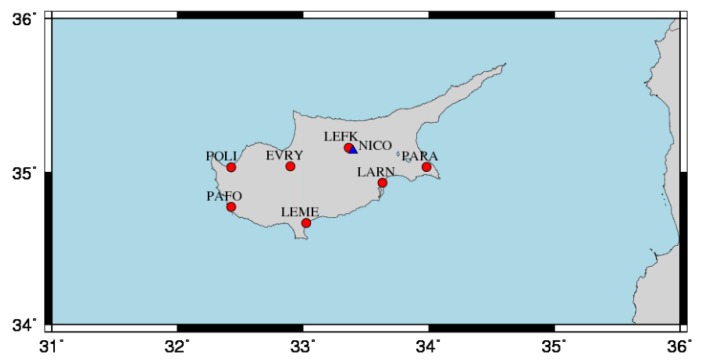
The permanent GNSS network of Cyprus (CYPOS, Cyprus Positioning System); red circles indicate the seven stations whose position time series are analyzed in this research; the blue triangle denotes the location of the NICO station which is an EPN (EUREF Permanent Network) site.

The main permanent GNSS station network in Cyprus is CYPOS, which has been established in 2008 and operated since by the Ministry of the Interiors Department of Lands and Surveys (DLS). The main objective of CYPOS is to support the needs of both DLS and the professional community of engineers with respect to surveying and geodetic applications, by means of single-base, network RTK and post-processing services. It consists of seven permanent, continuously operating GNSS reference stations installed at DLS buildings throughout the government-controlled areas of the country with inter-station distances of about 60 Km (see Figure 1). CYPOS stations are currently operating as Tier-3 CORS. The monumentation of GNSS antennas is comprised of stable stainless-steel structures (polar masts) located at specific locations at the rooftop of DLS buildings to achieve unobstructed satellite visibility. 

The detailed specifications and features of the seven permanent stations are illustrated in Table 1. It can be seen that all GNSS receivers can track GPS signals on the L1 (1575.42 MHz) and L2 (1227.60 MHz) bands and support GLONASS (GLObal NAvigation Satellite System) L1 (~1602 MHz) & L2 (~1246 MHz) sub-bands. Furthermore, all GNSS antennas are choke-ring type to minimize the effect of multipath, and they incorporate ultra-wideband Dorne-Margolin elements to optimize antenna gain.

In addition to CYPOS stations, there is a station of the European Permanent Network (EPN) with the identifier NICO located in Nicosia. NICO is a Tier-1 station operated by the German Federal Agency for Cartography and Geodesy (BKG). The station’s current hardware configuration is a Leica GR50 multi-GNSS receiver, supporting GPS, GLONASS, Galileo and BeiDou, and a Leica AR25 choke-ring antenna. NICO has been delivering GNSS observations since June 22nd, 1997.

### 2.2. Observation Dataset and Software

The GNSS observation dataset derived from CYPOS spans a time period of more than five years (30/11/2011–28/01/2017) and it was processed along with daily RINEX (Receiver Independent Exchange Format) data from 35 stations of EPN [33] including the NICO station. The computation of daily and multi-year solutions has been carried out using the Bernese GNSS software package, version 5.2 developed at the Astronomical Institute of the University of Bern (AIUB) [34]. Bernese is one of the reference software for precise GNSS computations adopted by major research institutes throughout the world.

Additional software developed at the Department of Geodesy and Surveying of the Aristotle University of Thessaloniki was used to compute specific quality metrics for the daily and multi-year GNSS solutions, as well as for the least-squares fitting of seasonal models to the position time series.

### 2.3. Computation of Daily Solutions

**Figure 2:**
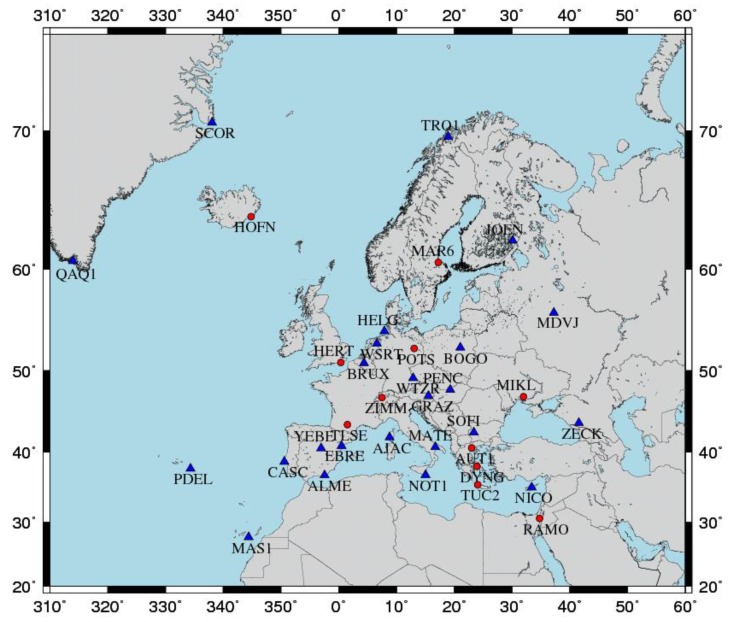
Map of EPN stations used throughout the GNSS data processing stage. The stations marked in blue represent the EPN reference stations used in the datum definition process.

The overall network that was formed for computing the daily solutions consists in total of 42 stations. Besides the seven (7) GNSS stations of CYPOS, the network includes 35 selected EPN stations whose geographical distribution is shown in Figure 2. Note that the stations marked in blue represent the reference stations which were employed for the datum definition in the daily and multi-year network solutions.

The processing of the daily GNSS observations in the aforementioned network was performed by the Bernese GNSS software, version 5.2, using the double-difference strategy in accordance to the EPN guidelines [33]. A summary of the general options that were adopted during the GNSS data processing is given in Table 2. The datum fixation for the daily solutions was implemented by the no-net-translation (NNT) condition to the known IGb08 coordinates of 24 EPN (Tier-1) stations (see Figure 2). The results from this processing step to be exploited in the computation of the multi-year solution consist of: (a) daily station positions and their respective formal errors, and (b) full covariance matrices of all daily network solutions. The number of participating stations in each daily solution ranges from a minimum of 27 up to a maximum of 42.

The number of formed baselines in the daily GNSS networks varied from 26 to 41. The baseline lengths range from 4 km to 3222 km, whereas the mean baseline length in the daily networks is 735 km. Depending on the baseline length, three different ambiguity resolution strategies were applied in our analysis (see Table 2). The average daily success rate of ambiguity resolution during the entire processing was 91%, with maximum and minimum values being 98% and 81%, respectively. The percentage of successfully resolved ambiguities over the time period of our analysis (11/2011–01/2017) showed a significant annual period. In particular, the maximum rates of resolved ambiguities occurred systematically during the winter, while the minimum success rates were obtained mostly in the summer period.

The daily adjustments of the GNSS network were performed in two different steps: the first one before the ambiguity resolution (free network solution) and the second one after introducing the resolved ambiguities as known parameters into the adjustment algorithm (fixed network solution). The median value of the daily formal errors (standard deviations) of the estimated station positions in the free network solution was 1.6 mm. The usage of the resolved ambiguities improved the precision of the estimated station positions in the fixed network solution. Specifically, the median of the daily formal errors was reduced to 0.8 mm for the X and Z components, and 0.4 mm for the Y component.

**Figure 3:**
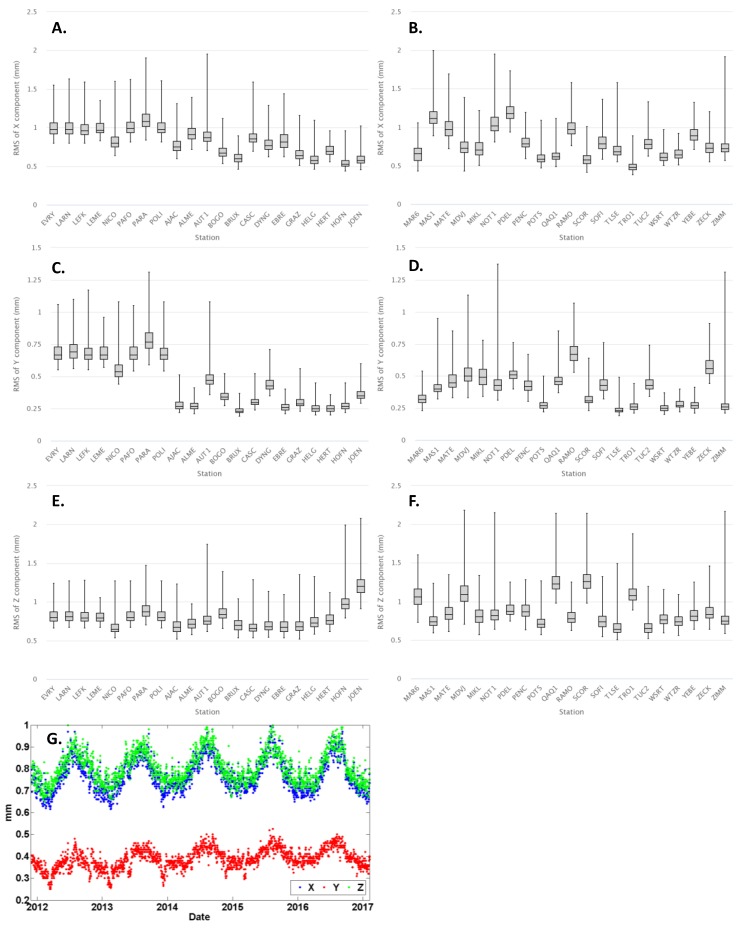
The box-plots (**A**,**B**), (**C**,**D**), (**E**,**F**) depict the range and median of the daily standard deviations respectively for the X, Y and Z components (mm) at each GNSS station, whereas the ninth plot (**G**) shows the temporal variation of the average daily precision for the estimated positions over all network stations.

To obtain a more insightful view on the results from the daily network adjustments, both the temporal and spatial variability of the formal precision for the estimated daily positions were analyzed. The results of these analyses are depicted in Figure 3 in the form of box-plots for the daily standard deviations related to the X, Y and Z components at each GNSS station, along with their daily median value over all stations for the individual X, Y and Z components. The latter values show a significant annual periodicity with their maxima appearing during the summertime and their minima during the winter (see Figure 3G). The CYPOS stations present the worst precision of the estimated daily positions in the Y component, which is almost two times worse with respect to the median value over all network stations (see Figure 3C,D). 

**Figure 4:**
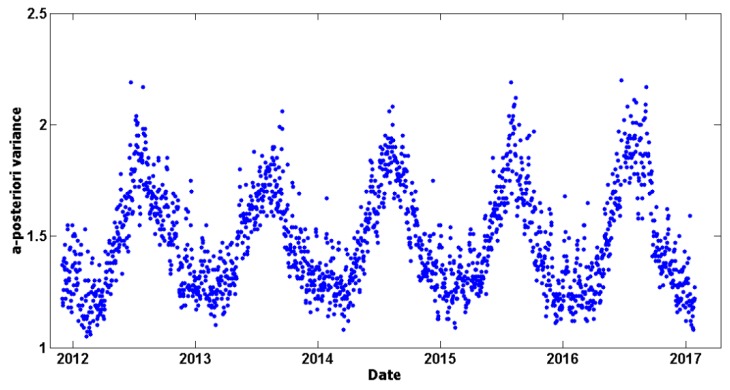
Daily a-posteriori variance factors derived by the adjustment of the GNSS network.

After computing each daily solution, a 7-parameter Helmert transformation was applied between the estimated daily positions and the official IGb08 positions at the 24 EPN (Tier-1) reference stations. This was carried out to detect ‘problematic’ reference stations that had to be excluded from the datum definition in the respective daily solutions. Such a screening process was iteratively implemented until no problematic reference stations were dictated by the post-fit residuals of the daily Helmert transformations. As an example of the quality metrics for the final daily solutions, the a-posteriori variance factor obtained by each daily network adjustment is illustrated in Figure 4. Its values show a significant annual periodicity which is explained by the fact that the variance factors are strongly correlated with the success rate of daily ambiguity resolution in the GNSS network. During the winter period the performance of ambiguity resolution is systematically better, thus leading to smaller adjusted residuals in the daily adjustments. On the other hand, in the summer period the adjusted residuals increase due to smaller percentages of successfully resolved ambiguities in the GNSS baselines, which is attributed to the lower accuracy of atmospheric models (mainly ionospheric) during that period.

### 2.4. Multi-Year Solution and Quality Assessment

After the computation of all daily GNSS solutions, a multi-year combined adjustment was performed based on the stacking of 1878 (unconstrained) daily normal equations (NEQ). The adopted reference frame of the multi-year solution is IGb08 and it was realized through the NNT condition on the positions and velocities of 24 EPN (Tier-1 and Tier-2) reference stations (see Figure 2). The reference epoch for the estimated positions was set to $t_{0}=2005.0$, identical to the reference epoch of the official IGb08 frame. The main products of interest from this step are the estimated positions (at $t_{0}$) and velocities of the CYPOS GNSS stations, along with their respective time series of position residuals.

The NEQ stacking procedure was applied iteratively in order to detect station discontinuities and data outliers. At each iteration step, the post-fit residuals were analyzed separately for each station using the FODITS (Find Outliers and Discontinuities In Time Series) module of the Bernese 5.2 software. Two iterations were enough for identifying a total of 11 station discontinuities and 16 data outliers. The official list of EPN station coordinates (www.epncb.oma.be/_productsservices/coordinates) includes six more discontinuity events that are relevant to this network configuration for the considered time period (11/2011–01/2017)–these were also taken into account in the final computation of the multi-year solution. Note that 15 out of 17 station discontinuities were not caused by geophysical events but they are attributed to GNSS receiver or antenna change at 12 EPN stations. The other two discontinuities occurred at CYPOS stations (LEFK, PAFO) for which particular mention is given in the following sections of this paper. All station discontinuities were modeled during the multi-year adjustment by applying tight relative constraints (σ = 0.01 mm/yr) to the estimated velocities before and after the respective discontinuity epoch.

**Figure 5:**
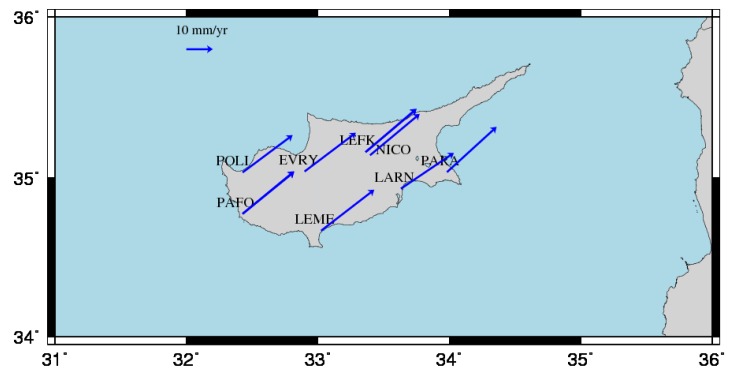
Estimated horizontal velocities of the CYPOS stations in the IGb08 frame.

The final estimates of positions and velocities at the CYPOS stations are summarized in Table 3 and Table 4 respectively. The stations LEFK and PAFO appear with multiple estimates in their respective results due to the detected discontinuities mentioned previously. The horizontal components of the estimated velocities are plotted in Figure 5. The vertical component of the estimated velocities is mostly negligible (<1 mm/year), with the exception of LARN and, to a lesser extent, PAFO. The first of these stations appears to have a significant downward trend of about 5 mm/year which needs to be further investigated regarding its cause.

**Figure 6:**
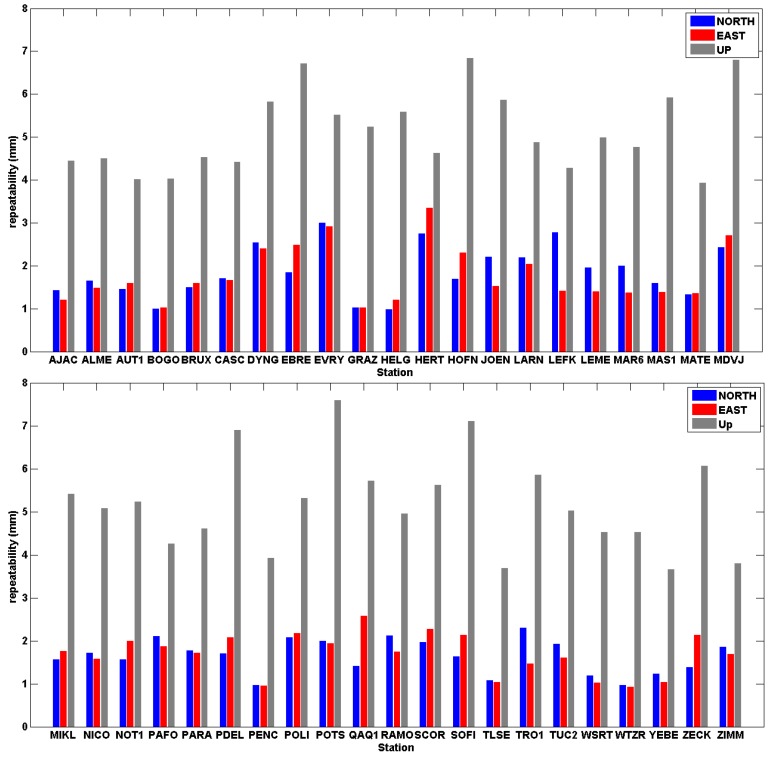
The root-mean-square (RMS) of the coordinate differences between the daily normal equation (NEQ) solutions and the multi-year solution in IGb08 for each position component (north, east, up).

To give a general overview of the quality of the multi-year solution, the coordinate repeatability for all network stations is depicted in Figure 6. Specifically, the root-mean-square (RMS) of the daily residuals is shown for each station (separately for north, east and up component) as obtained from the least-squares NEQ stacking adjustment–these values are less than 1 cm for all stations. The ‘up’ component has 2–3 times larger values than the horizontal components, as usually expected. Note that a significant part of the daily residuals at the CYPOS stations is attributed to annual periodic signals due to unmodeled loading effects in the GNSS data (see Section 4).

**Figure 7:**
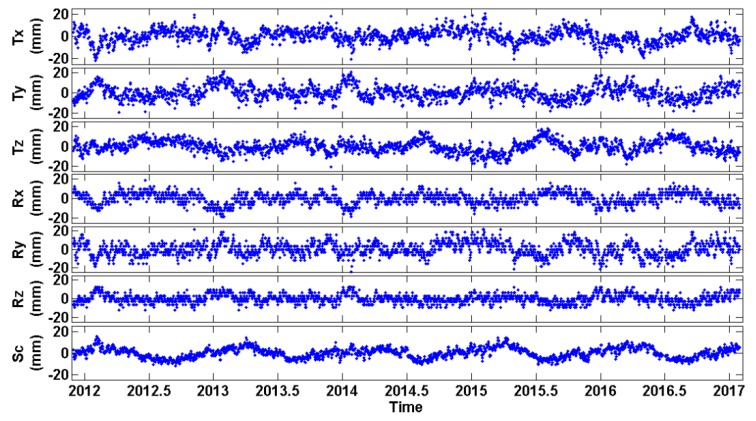
Estimated Helmert parameters (three Translations, three Rotations and Scale) between the recomputed daily solutions and the multi-year solution.

**Figure 8:**
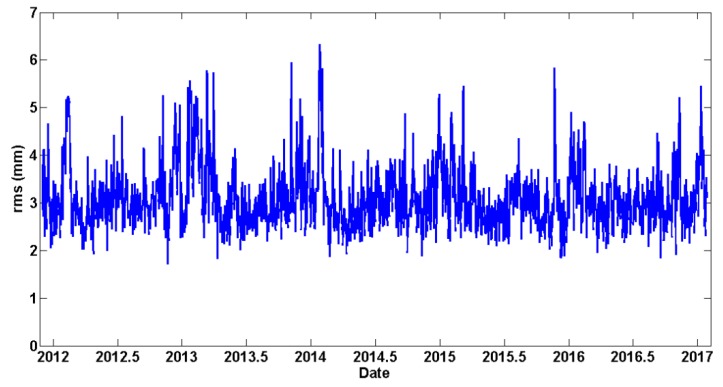
RMS of Helmert transformation residuals between the re-computed daily solutions and the multi-year solution.

As an additional validation for the multi-year solution, the daily coordinates of all network stations have been re-computed in IGb08 via minimally constrained adjustments of the original daily NEQ. The estimated positions and velocities of the 24 EPN reference stations (as obtained from the multi-year solution) were used to provide the ‘daily reference coordinates’ for the NNT datum condition in all of these adjustments. The square root of the a-posteriori variance factor of the re-computed daily solutions varied between 1–1.5 mm. All of the re-computed daily solutions were compared with the multi-year solution by a simple least-squares fit using the 7-parameter Helmert transformation. The estimated Helmert parameters of these daily fits are shown in Figure 7, whereas the total RMS of the post-fit daily coordinate residuals is illustrated in Figure 8.

To verify the correctness of these results, the differences between the estimated velocity values from the multi-year solution and the official velocity values provided by EPN (both of these sets are expressed in the IGb08 frame) were computed. This comparison was obviously applied only to the EPN stations which were included in the network. The mean of these velocity differences is close to zero (<0.1 mm/yr) for all three topocentric components, whereas their dispersion for the north, east and up component is 0.6 mm/yr, 0.4 mm/yr and 0.5 mm/yr respectively. Such differences were expected and are mainly due to the different time span of the used GNSS data (~5 years in our case vs. 20 years in the EPN case) and the different stacking strategies that were employed in the respective multi-year solutions (NEQ stacking in our case vs. time-series stacking in the EPN case).

## 3. Horizontal and Vertical Secular Displacements in Cyprus

Based on the estimated velocities in the CYPOS network, it is deduced that Cyprus behaves as a stable area in terms of horizontal tectonic movements. All stations seem to follow similar horizontal displacements in the IGb08 frame, at a rate of about 2.5 cm/yr along a northern-eastern (NE) direction (see Figure 5). In addition, the uniform horizontal motion of the CYPOS stations allows the use of the standard kinematic model of plate tectonics, namely Euler’s theory for rigid motion on spherical bodies [5,26,35], in order to perform further analysis of the estimated station velocities. This is useful in our study not only for estimating the overall motion of Cyprus on the Earth’s surface, but also for identifying its kinematic ‘congruence’ with respect to neighboring tectonic plates.

The theory of plate tectonics provides a unified framework for understanding the mechanisms of global geodynamic processes. Its rationale relies on the hypothesis that the surface of the Earth is divided into a number of rigid blocks (tectonic plates) which remain in relative secular motion according to Euler’s fixed-point theorem [26,35]. More specifically, the rigid motion of any tectonic plate on the Earth’s spherical surface is modeled as a rotation around an axis passing though the center of the Earth, whose angular velocity corresponds to the so-called Euler vector. The latter is described by a triplet of basic parameters, namely the Euler pole coordinates (latitude, longitude) which describe the direction of the rotational axis with respect to the Earth’s surface, and the magnitude of the angular velocity vector. Their determination from either geological and geophysical data [27,35] or modern geodetic data [1,2,4,28,36] has led to the development of various plate motion models which have contributed significantly to better understanding of global tectonics.

By presuming that Cyprus represents a rigid crustal block, the three Euler parameters of its secular motion have been determined by a simple least-squares procedure (see [37]) using as observables the horizontal velocities in the CYPOS network. The respective estimates of the Euler pole position (Φ, Λ) and the anti-clockwise angular velocity (ω) are given in Table 5. Note that the large values of formal estimation errors for Φ and Λ are due to the inherent instability in the least-squares adjustment caused by the limited area of the CYPOS GNSS stations on the Earth’s surface.

The values of the post-fit velocity residuals from the aforementioned adjustment ranged from −0.2 mm/yr to 0.7 mm/yr for the East component, and from −0.3 mm/yr to 2.1 mm/yr for the North component, with a total RMS over the entire network of 0.9 mm/yr. Overall, the velocity residuals were consistently smaller than 1 mm/yr, with the exception of the GNSS station located in Larnaca (LARN) which showed residuals of 2.1 mm/yr and 0.5 mm/yr for the North and East component, respectively. This particular station may be affected by localized effects, mainly the underlying geology, which likely influence its observed GNSS time series and the respective velocity estimates from the multi-year network solution. Note that the area where the building was constructed was previously part of nearby lake.

**Figure 9:**
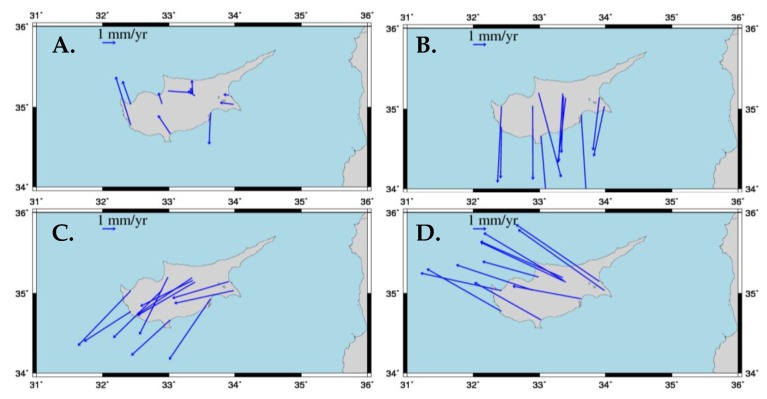
Horizontal velocities at the CYPOS stations with respect to different ‘plate-fixed’ frames: (**A**) Anatolia, (**B**) Arabian, (**C**) African, (**D**) Eurasia.

In order to detect the congruence of Cyprus’ horizontal motion with that induced by each of its neighboring plates (Africa, Arabia, Anatolia, Eurasia), the Actual Plate Kinematic and Crustal Deformation Model (APKIM2005) [36] was used to reduce the IGb08 estimated velocities of the CYPOS stations to each of the respective plates. In this way, horizontal velocities with respect to different ‘plate-fixed’ frames were obtained, which serve as a simple diagnostic tool to assess Cyprus’ neotectonic crustal movement. Note that APKIM2005 is a geodetically-derived model based on the complete global set of ITRF2005 station velocities, and it consists of Euler rotation vectors for 17 major plates over the Earth. The APKIM2005 based Euler pole parameters for the four plates of interest are listed in Table 6. Two basic steps were followed to obtain plate-fixed horizontal velocities of the CYPOS stations: (a) first, the IGb08 estimated velocities were transformed to ITRF2005, and (b) the contribution of each plate’s rotational motion was subtracted from the transformed ITRF2005 velocities to obtain residual ‘plate-fixed’ velocities. The final results are depicted in Figure 9, whereas the statistics of the residual velocities for each case are listed in Table 7. From these results it is safely confirmed that Cyprus does not belong in either of the Arabian, African and Eurasia plates. Indeed, the residual plate-fixed velocities in these cases show a systematic pattern of several mm/yr. On the other hand, the horizontal motion of the CYPOS stations seems to be more consistent with the rotational motion of the Anatolia plate, as the residual plate-fixed velocities behave in a more random-like pattern at an average rate that is smaller than 1 mm/yr. 

Regarding the secular vertical displacements at the CYPOS stations, it is not quite safe to reach final conclusions as the time span of their GNSS time-series is rather small (~5 years) for estimating reliable vertical velocities. The vertical motion for most stations appears to be close to zero and in the range of the GNSS data processing errors. To be more specific, and excluding the stations LARN and PAFO, the rates of vertical displacements in Cyprus vary from −0.4 mm/yr at the station POLI to 0.6 mm/yr at PARA. On the other hand, LARN and PAFO seem to experience totally different vertical motions relative to the other stations. The station LARN exhibits a downward motion at a rate of −4.9 mm/yr, whereas PAFO shows an uplift at a rate of 1.7 mm/yr. For those stations special care should be given in future projects in order to investigate the true nature of their dynamic behavior.

## 4. Analysis of Position Time Series at CYPOS Stations

### 4.1. Station Discontinuities

During the computation of the multi-year solution, the time series of daily residuals at each network station were examined to identify possible discontinuities. Two different strategies were applied for this purpose: the first relied on the use of the FODITS script of the Bernese GNSS software [23], whereas the second employed a modified version of the ‘step detection algorithm’ [38], which is based on the comparison between the magnitude of estimated ‘steps’ in the time series and a predefined threshold value (either 2.5 times the formal daily coordinate precision or 3 mm). Both strategies managed to identify the same number of discontinuities over the network: nine at EPN stations, and two at CYPOS GNSS stations.

The detected discontinuities in the residual position time series of stations refer to: PAFO (10/03/2016) and LEFK (22/03/2013). It should be noted that none of these stations had any GNSS receiver or antenna change during the examined time period. The discontinuity at the PAFO station is rather small and not visually detectable. Its magnitude is 3.4 mm, 3.0 mm and 5.4 mm for the North, East and Up components, respectively. The discontinuity at the LEFK station is larger and it affects mostly the East component (17.8 mm), whereas the North and Up components are both influenced to a lesser extent by −2.3 mm and 6.8, respectively. It is worth mentioning that the NICO station (which belongs both to EPN and IGS networks) is located approximately 6 km from the LEFK station, and it does not appear to have any similar offset in its position time series at the same epoch. Hence, the discontinuity at the LEFK station should not probably be linked to local geodynamical activity, but it is likely related to unknown hardware failure for this particular station.

### 4.2. Estimation of Periodic Signals

After the computation of the multi-year solution, the residual time series of daily positions were formed for all network stations. The focus in this section relies on the temporal behavior (of the topocentric components) of these residual time series at the seven CYPOS stations. Their rms values range from 1.7 mm up to 3 mm for the horizontal components, and from 4.6 mm up to 5.5 mm for the vertical component. Only a portion of these values should be attributed to GNSS data noise, since a large part of the temporal variation in the residual time series is related to systematic effects caused by unmodeled geodynamical processes (non-tidal loading effects).

All permanent GNSS stations in Cyprus show strong annual signals in their position time series, both for the horizontal and vertical components. Such signals originate mostly from unmodeled non-tidal Earth loading effects, and they have a significant impact (of several mm) on the daily station positions. Their characteristic parameters (amplitude, phase) were separately estimated for each topocentric component through a least-squares fit of a simple sinusoidal model to the residual time series of each station. The sinusoidal function that was initially used in these tests contained a mixture of annual and semi-annual periodic terms. However, the estimated parameters of the semi-annual signals were found to be statistically negligible for all stations and, thus, another series of least-squares fittings was applied using only annual periodic terms. The respective estimates of the characteristic parameters of the annual signals at the seven CYPOS stations are given in Table 8.

According to Table 8, the amplitudes of the annual signals vary from 0.6 mm to 4.1 mm. The highest value occurs in Up component at the station located in Limassol (LEME), whereas the same station has the smallest amplitude in the East component (0.6 mm). Note, however, that the daily GNSS data from this particular station cover only a two-year period (2015–2017) and, thus, the estimated annual signals of its topocentric components will not be as accurate compared to the other CYPOS stations – this may also explain the large difference in the estimated phase value for LEME with respect to the rest of the stations. Finally, the stations EVRY and LARN exhibit the highest amplitudes for the annual periodic variation in horizontal position amongst all other stations. 

**Figure 10:**
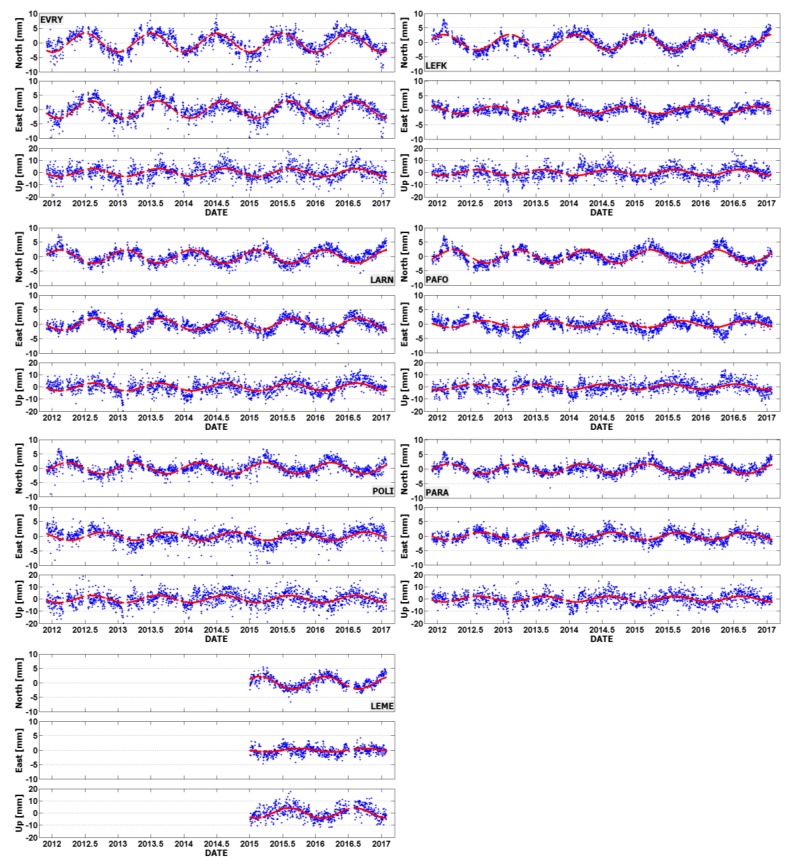
Residual position time series of the CYPOS stations: EVRY, LEFK, LARN, PAFO, POLI, PARA and LEME.

A series of plots showing the residual position time series with the fitted annual curves at the CYPOS GNSS stations is given in Figure 10. The magnitude of the daily post-fit residuals (i.e., after the removal of the estimated annual signals) remains almost the same in all CYPOS stations. Their rms varies between 1–2 mm for the East and North components, and between 4–5 mm for the Up component. Interestingly enough, the temporal behavior of the post-fit residuals is not completely random, but it was found to contain additional small signals with periods lower than 90 days. 

### 4.3. Comparison with Geophysical Loading Models

It is well-known that a significant part of the nonlinear variability in GNSS position time series originates from seasonal variations due to unmodeled loading effects on the Earth’s crust [12,14,15]. To identify the extent of such Earth loading signals within the residual position time series at the CYPOS stations, time series of ground displacements from a variety of geophysical loading models have been computed. 

Specifically, the following sources have been used in this analysis: (a) the global Land Surface Discharge Model (LSDM) from the Deutsches GeoForschungsZentrum (GFZ) to obtain the hydrological loading component due to continental water storage (CWSL), (b) the surface pressure model from the European Centre for Medium-Range Weather Forecasts (ECMWF) to obtain the non-tidal atmospheric loading component (NTAL), (c) the Max-Planck Institute ocean model (MPIOM) to obtain the non-tidal ocean loading component (NTOL), and lastly (d) the GFZ model of barystatic sea-level variations to obtain the sea-level loading component (SLEL). Both horizontal and vertical components of loading displacements were computed, at daily resolution, from the aforementioned geophysical models at six CYPOS stations, namely EVRY, LARN, LEFK, PARA, PAFO and POLI. All computations covered the same time period using the available GNSS data from 30/11/2011 to 28/01/2017–the station LEME was excluded due to its limited time span. It is noted that all loading displacements have been computed with respect to a global centre-of-figure (CF) frame, so that they can be consistent with the GNSS-derived results.

**Figure 11:**
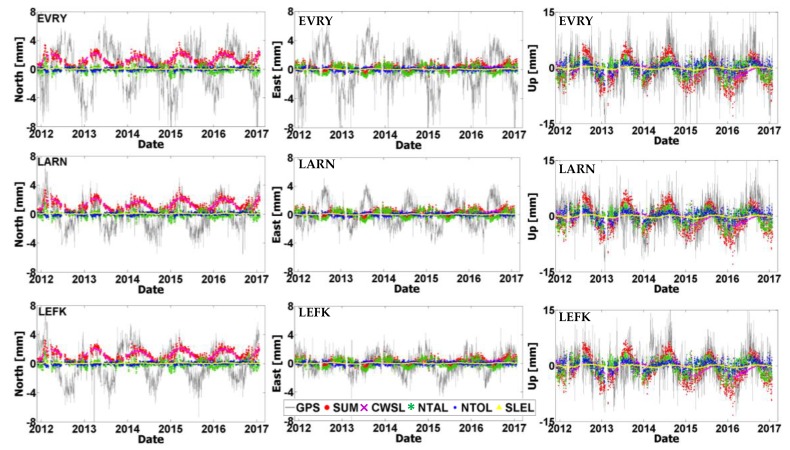
Plots showing the time series of GNSS-based residual positions (in grey) and the loading displacements originating from CWSL (Continental Water Storage Loading, purple crosses), NTAL (Non-Tidal Atmospheric Loading, green stars), NTOL (Non-Tidal Ocean Loading, blue squares) and SLEL (Sea-LEvel Loading, yellow triangles). The total loading effect is also depicted in the above plots (red dots). The results refer to the stations EVRY, LARN and LEFK (Note: Scale for UP component is larger).

**Figure 12:**
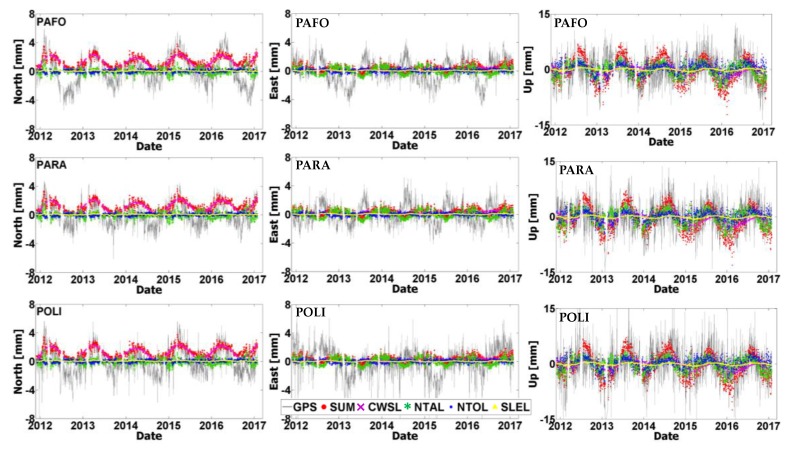
Plots showing the time series of GNSS-based residual positions (in grey) and the loading displacements originating from CWSL (Continental Water Storage Loading, purple crosses), NTAL (Non-Tidal Atmospheric Loading, green stars), NTOL (Non-Tidal Ocean Loading, blue squares) and SLEL (Sea-LEvel Loading, yellow triangles). The total loading effect is also depicted in the above plots (red dots). The results refer to the stations PAFO, PARA and POLI (Note: Scale for UP component is larger).

The comparison between the time series of loading displacements and the GNSS-based residual position time series at the CYPOS stations is depicted in Figure 11 and Figure 12. Both the individual loading effects and their total sum are displayed in these figures, separately for each topocentric component. The magnitude of the combined loading effect does not vary among the different stations (due to the small geographical region covered by the CYPOS stations), and it is significantly stronger in the Up component compared to the horizontal components. Specifically, the RMS of the total loading displacements throughout the time period 30/11/2011–28/01/2017 reaches 0.7 mm, 0.5 mm and 3.3 mm for the North, East and Up component, respectively, see also Table 9.

In terms of relative importance of the individual loading effects, the non-tidal atmospheric loading (NTAL) has the largest contribution to the vertical and East components of daily displacements at the CYPOS stations. On the other hand, the continental water storage loading (CWSL) seems to be mostly significant for the North component of the horizontal daily displacements, and it affects to a lesser extent the other topocentric components as well. The other two loading sources (NTOL, SLEL) have relatively smaller contribution to the daily displacements of the CYPOS stations.

## 5. Conclusions

The following conclusions can be drawn based on the numerical results of this paper:

- the area of Cyprus seems to be stable, without suffering any notable local crustal deformations, at least within the time range considered in the present study (11/2011–01/2017);
- the GNSS station that is located in Larnaca (LARN) shows some significant local effects, both in the horizontal and vertical components of its position time series. It is surmised that these effects are attributed to the underlying geology;
- the horizontal and vertical positions of all CYPOS GNSS stations have annual periodic variations of considerable magnitude (several mm)—the semi-annual periodic displacements however were found to be negligible;
- some of the CYPOS GNSS stations appear to have periodic variations in their spatial positions at higher frequencies (120 to 60 days)—the related results have not been presented herein (due to space limitations) and they need to be investigated in detail in the future.

The analysis that was presented in this study will be further continued with the assimilation of additional data from the CYPOS GNSS stations (post-2017) and the implementation of extra processing options (use of the IGS14 reference frame instead of IGb08). Furthermore, the colored noise characteristics in the derived coordinate time series, in terms of temporal and spatial correlations, will be investigated in future work.

## Figures and Tables

**Table 1:** The permanent GNSS stations of the CYPOS (Cyprus Positioning System) network.

Station ID	Location	Receiver Model	Antenna Model	Supported GNSS
EVRY	Evrychou	Leica GRX1200+ GNSS	Leica AR25	GPS + GLONASS
LARN	Larnaca	Leica GMX902 GG	Leica AT 504 GG	GPS + GLONASS
LEFK	Nicosia	Leica GMX902 GG	Leica AT 504 GG	GPS + GLONASS
LEME	Limassol	Leica GMX902 GG	Leica AT 504 GG	GPS + GLONASS
PAFO	Paphos	Leica GMX902 GG	Leica AT 504 GG	GPS + GLONASS
PARA	Paralimni	Leica GMX902 GG	Leica AT 504 GG	GPS + GLONASS
POLI	Polis	Leica GRX1200+ GNSS	Leica AR25	GPS + GLONASS

**Table 2:** GNSS daily data processing parameters and settings.

Parameter	Setting
Basic Observable	GNSS carrier phase. Code-only for receiver clock sync and ambiguity resolution. Melbourne-Wübbena wide lane combination.
Elevation Cut-Off Angle	10°, elevation-dependent weighting (cosz).
Data Sampling	30 s and 180 s in final solution.
Modeled Observable	Ionosphere-free linear combination of double-differenced carrier phase.
Ground/Satellite APC calibration	Absolute Antenna Phase Center (APC) corrections (igs08.atx).
Tidal Displacements	IERS 2010 conventions (solid Earth tides) FES2004 conventions (ocean loading corrections) No atmospheric loading corrections
Orbits and Earth Rotation Parameters (ERPs)	IGS Final GPS and GLONASS orbits and ERPs
Ionosphere	First-order ionospheric delays eliminated by forming ionosphere-free L1/L2 linear combination. Higher-order ionospheric corrections are applied. Regional ionospheric maps were used to increase the number of resolved ambiguities in Quasi-Ionosphere Free (QIF), L5/L3 and L1/L2 ambiguity resolution.
Ambiguity Resolution	Ambiguities are resolved in a baseline-by-baseline mode: - Melbourne-Wübbena approach (<6000 Km) - Quasi-Ionosphere-Free (QIF) approach (<2000 Km) - Phase-based Wide Lane/ Narrow Lane (<200 Km) - Direct L1/L2 method, also for GLONASS (<20 Km) - GLONASS is considered for ambiguity resolution (<2000 Km)
Troposphere	Dry GMF (prior model), estimation of hourly zenith delay corrections for each station using Wet GMF. Horizontal gradient parameter estimated each day per station (Chen-Herring)
Reference Frame	IGb08, no-net translation on reference station coordinates and velocities (IGb08.snx)

**Table 3:** Estimated positions of the CYPOS stations in IGb08 at epoch $t_{0}=2005.0$.

Station ID	$X\;(t_{0})$	$Y\;(t_{0})$	$Z\;(t_{0})$
EVRY	4389846.035	2839909.319	3641645.008
LARN	4358623.310	2899369.048	3631599.949
LEFK ^1^	4360035.737	2870860.968	3652605.816
	4360035.736	2870860.987	3652605.816
LEME	4403058.471	2862122.638	3607630.266
NICO	4359415.715	2874117.069	3650777.829
PAFO ^1^	4427028.128	2812497.092	3617359.846
	4427028.124	2812497.091	3617359.841
PARA	4335378.631	2922300.281	3641064.127
POLI	4413130.062	2803627.159	3640911.041

^1^ LEFK and PAFO have multiple estimated positions due to associated discontinuities on 21/3/2013 and 9/3/2016, respectively.

**Table 4:** Estimated velocities (mm/yr) of CYPOS stations in IGb08.

Station ID	$V_{north}$	$V_{east}$	$V_{up}$
EVRY	14.7	19.5	0.2
LARN	13.6	20.2	−4.9
LEFK ^1^	16.3	19.3	0.1
	16.4	19.3	0.2
LEME	15.6	20.3	0.3
NICO	15.7	18.9	−0.3
PAFO ^1^	16.1	19.6	1.7
	15.9	19.7	1.6
PARA	17.2	18.9	0.6
POLI	14.2	19.1	−0.4

^1^ LEFK and PAFO have multiple estimated velocities due to associated discontinuities on 21/3/2013 and 9/3/2016, respectively.

**Table 5:** Estimates of Euler pole parameters for Cyprus in the IGb08 frame.

Φ [deg]	Λ [deg]	ω [deg/Myear]
49.83 ± 33.98	13.19 ± 15.30	0.629 ± 0.036

**Table 6:** Euler pole parameters for different tectonic plates according to Actual Plate Kinematic and Crustal Deformation Model (APKIM2005) [36].

	Φ [deg]	Λ [deg]	ω [deg/Myear]
Anatolia	40.0 ± 0.2	28.3 ± 0.4	2.021 ± 0.137
Arabian	49.5 ± 0.8	4.8 ± 3.3	0.596 ± 0.029
African	49.3 ± 0.4	280.5 ± 1.0	0.273 ± 0.002
Eurasia	54.5 ± 0.4	262.9 ± 0.5	0.258 ± 0.001

**Table 7:** Statistics of horizontal velocities at the CYPOS stations with respect to different ‘plate-fixed’ frames. All values are given in mm/yr.

	Anatolia	Arabian	African	Eurasia
***North component***				
Mean	0.7	−5.1	−2.9	2.7
RMS	1.6	5.2	3.1	3.0
***East component***				
Mean	−0.3	0.0	−3.7	−5.8
RMS	0.9	0.7	3.8	5.8

**Table 8:** Estimated parameters of annual signals in the position time series of CYPOS GNSS stations.

Station ID	Amplitude [mm]			Phase [deg]		
	North	East	Up	North	East	Up
EVRY	3.2	3.0	3.2	114.5	80.9	74.0
LARN	2.3	2.1	3.1	253.0	62.2	71.9
LEFK	2.7	1.3	2.4	259.5	344.9	80.6
LEME	2.1	0.6	4.1	45.2	190.0	244.8
PAFO	2.3	1.2	2.2	211.6	49.0	109.0
PARA	1.7	1.3	2.3	233.0	59.9	78.8
POLI	2.0	1.4	2.9	212.7	22.2	79.4

**Table 9:** RMS of the (a) GNSS-based residual position time series and (b) daily displacements due to total loading (CWSL+NTAL+NTOL+SLEL) at the CYPOS stations. All values are given in mm.

Station ID	GNSS-Based			Total Loading		
	North	East	Up	North	East	Up
EVRY	2.9	2.7	4.9	0.7	0.5	3.3
LARN	2.0	1.7	4.3	0.7	0.5	3.3
LEFK	2.4	1.5	4.1	0.7	0.5	3.3
PAFO	2.0	1.7	3.9	0.8	0.5	3.2
PARA	1.5	1.4	4.0	0.7	0.5	3.3
POLI	1.8	1.9	4.7	0.7	0.5	3.2
